# High Glycemic Diet Is Related to Brain Amyloid Accumulation Over One Year in Preclinical Alzheimer's Disease

**DOI:** 10.3389/fnut.2021.741534

**Published:** 2021-09-27

**Authors:** Matthew K. Taylor, Debra K. Sullivan, Jill K. Morris, Eric D. Vidoni, Robyn A. Honea, Jonathan D. Mahnken, Jeffrey M. Burns

**Affiliations:** ^1^Department of Dietetics and Nutrition, University of Kansas Medical Center, Kansas, KS, United States; ^2^University of Kansas Alzheimer's Disease Center, Fairway, KS, United States; ^3^Department of Neurology, University of Kansas Medical Center, Kansas, KS, United States; ^4^Department of Biostatistics, University of Kansas Medical Center, Kansas, KS, United States

**Keywords:** high glycemic diet, amyloid, alzheimer's disease, glycemic load, sugar

## Abstract

**Objective:** To test the hypothesis that high glycemic diet is related to 1-year change in brain amyloid based on our prior cross-sectional evidence that high glycemic diet is associated with brain amyloid.

**Methods:** This longitudinal, observational study assessed the relationship between reported habitual consumption of a high glycemic diet (HGDiet) pattern and 1-year brain amyloid change measured by Florbetapir F18 PET scans in 102 cognitively normal older adults with elevated or sub-threshold amyloid status that participated in a 1-year randomized, controlled exercise trial at the University of Kansas Medical Center in Kansas City.

**Results:** Among all participants (*n* = 102), higher daily intake of the HGDiet pattern (β = 0.06, *p* = 0.04), sugar (β = 0.07, *p* = 0.01), and total carbohydrate (β = 0.06, *p* = 0.04) were related to more precuneal amyloid accumulation. These relationships in the precuneus were accentuated in participants with elevated amyloid at enrollment (*n* = 70) where higher intake of the HGDiet pattern, sugar, and carbohydrate were related to more precuneal amyloid accumulation (β = 0.11, *p* = 0.01 for all measures). In individuals with elevated amyloid, higher intake of the HGDiet pattern was also related to more amyloid accumulation in the lateral temporal lobe (β = 0.09, *p* < 0.05) and posterior cingulate gyrus (β = 0.09, *p* < 0.05) and higher sugar and carbohydrate intake were also related to more amyloid accumulation in the posterior cingulate gyrus (β = 0.10, *p* < 0.05 for both measures).

**Conclusion:** This longitudinal observational analysis suggests that a high glycemic diet relates to higher brain amyloid accumulation over 1 year in regions of the temporoparietal cortex in cognitively normal adults, particularly in those with elevated amyloid status. Further studies are required to assess whether there is causal link between a high glycemic diet and brain amyloid.

**Clinical Trial Registration:** ClinicalTrials.gov, Identifier (NCT02000583).

## Introduction

Cerebral accumulation of amyloid-β plaque is a classic pathological hallmark of Alzheimer's disease (AD). Cerebral amyloid is present in the brain years before the onset of symptomatic dementia and has been hypothesized to be an early initiator of the AD process (1, 2) although amyloid's role as either cause or consequence of AD remains unclear and other etiologic hypotheses have been proposed (3). Nonetheless, evidence suggests that cerebral amyloid accumulation is an important signal for increased risk of developing symptomatic AD (4) in the 20–40% of cognitively unimpaired older adults who meet the research definition of “preclinical AD” due to having elevated cerebral amyloid (5).

Clinical studies increasingly link metabolic dysfunction, both centrally and peripherally, with AD. Reduced brain metabolism is a long-recognized hallmark of AD that occurs prior to dementia symptoms (6, 7). Impairments in systemic metabolism are linked to central nervous system metabolism as individuals with impaired peripheral glucose metabolism (elevated glucose, insulin resistance, or type two diabetes) exhibit reduced brain glucose uptake (8) and have higher risk of cognitive decline and neurodegeneration (9). Reductions in cerebral glucose metabolism have also been purported to increase brain amyloid accumulation (10). Upstream of metabolic impairment are behavioral factors, such as diet and exercise, that likely contribute to or counteract metabolic perturbations and may influence AD risk. There has recently been a great deal of emphasis placed on these potentially modifiable factors.

Our group previously reported a cross-sectional relationship between high glycemic diet, a diet high in refined carbohydrate and sugar, and cerebral amyloid levels in cognitively normal older adults that underwent amyloid PET scans to screen for eligibility for our Alzheimer's Prevention through Exercise (APEx) clinical trial (11). Based on these data, we hypothesize that a high glycemic diet influences AD pathology, possibly through its influence on metabolic factors that may play a role in AD (12). Here, we tested our hypothesis that high glycemic diet influences 1-year change in amyloid deposition in cognitively normal older adults with elevated or sub-threshold amyloid status that completed the APEx clinical trial.

## Methods

This secondary analysis was conducted using data from the APEx trial (NCT02000583) (13) at the University of Kansas Alzheimer's Disease Center (KU ADC). APEx was a randomized, controlled trial that assessed the effect of 1 year of aerobic exercise (150 min/week) vs. standard of care education on cerebral amyloid measured by florbetapir PET.

### Participants

**Figure 1:**
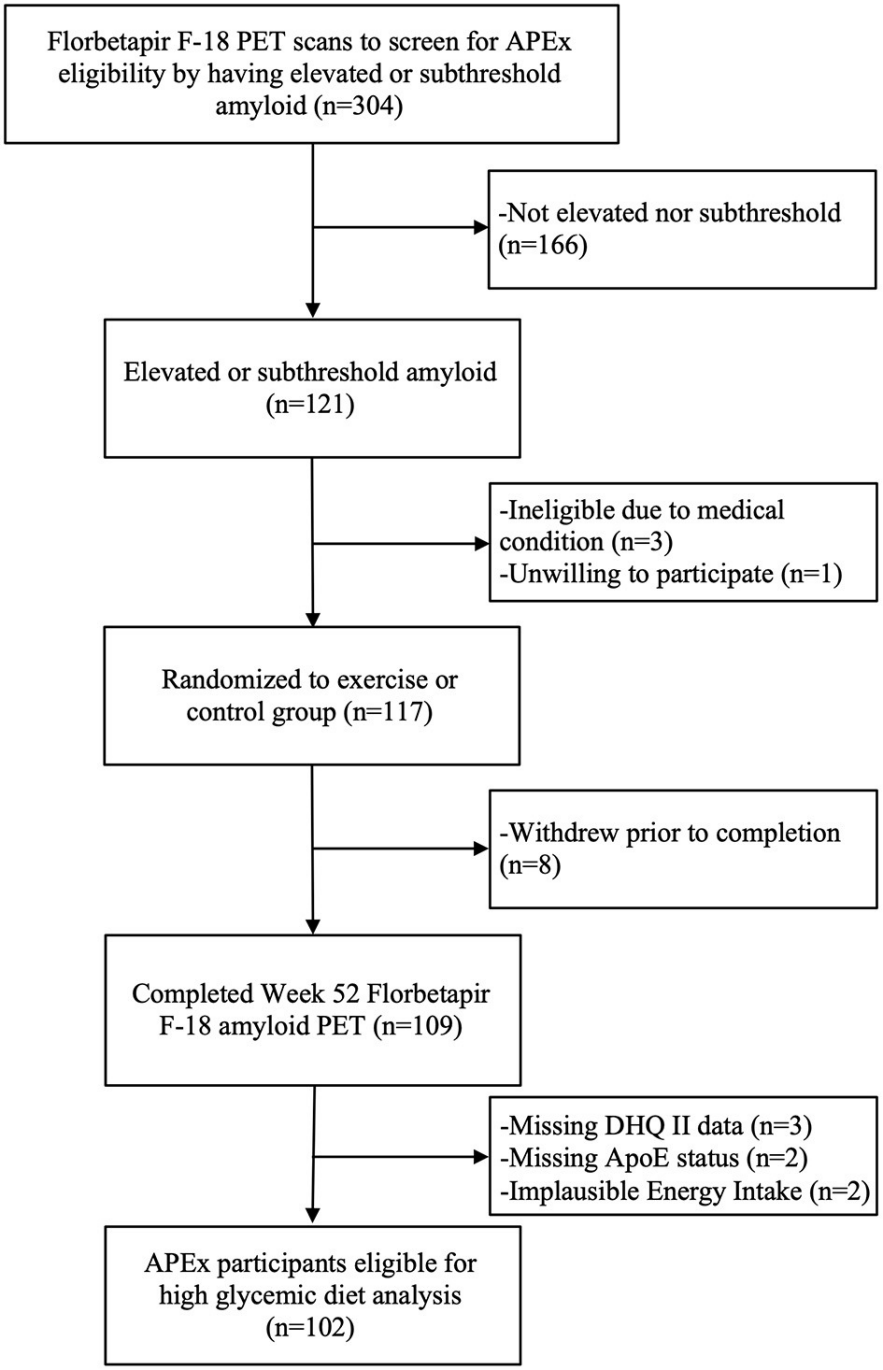
Diagram of the flow of participants included in this analysis.

Data from 106 APEx participants randomized to the exercise intervention (*n* = 73) or control group (*n* = 33) were available for this analysis. We excluded *n* = 2 participants for implausible reported daily energy intake (females: <500 or >3,500 kcals, males: <800 or >4,000 kcals) (14) and *n* = 2 participants for missing APOE status. The APEx study enrolled a sample enriched with individuals who had elevated cerebral amyloid while remaining cognitively normal. Enrollment occurred between March 1, 2014 and October 31, 2018. Participants were eligible to enroll in the APEx study if their amyloid screening PET scans revealed “elevated” or “sub-threshold” cerebral amyloid (individuals with non-elevated cerebral amyloid just below the cutoff). Additional inclusion criteria included age ≥65 years, being cognitively normal with a Clinical Dementia Rating (CDR) score of 0 (15), being sedentary or underactive based on the Telephone Assessment of Physical Activity (16) (score of ≤4), having stable medications for 30 days, and being physically healthy enough to partake in a 1-year aerobic exercise intervention. A trained clinician performed the CDR assessment. Assessments were reviewed at the KU ADC's consensus diagnosis conference to exclude those with MCI or dementia syndromes. Potential participants were excluded from APEx for presence of clinically meaningful depression or anxiety, insulin-dependent diabetes, uncontrolled hypertension, or recent history of major neuropsychiatric, musculoskeletal, or cardiorespiratory impairment in the past 2 years. The flow of participant inclusion in this data analysis is illustrated in Figure 1. The study protocol was approved by the Institutional Review Board at the University of Kansas Medical Center and informed consent was obtained from all study participants according to institutional guidelines.

### Description of Exercise Intervention

Study participants were randomized (2:1 ratio) to either an exercise intervention of 150 min per week of supervised, community-based aerobic exercise for 52 weeks or the control group. Those randomized to the control group did not receive supervised exercise, yet received education regarding the importance of exercise based on information from the National Institute on Aging (17).

### Anthropometric Measures

Body weight was measured with a calibrated scale (±0.1 kg) and height was measured with a wall-mounted stadiometer. BMI (kg/m^2^) was calculated from participant weight and height measurements.

### Dietary Intake Assessment

Baseline measurement of usual dietary intake was implemented within the APEx clinical trial to conduct planned secondary analyses investigating diet's relationship with AD risk. Participants completed the web-based National Cancer Institute's (NCI) Diet History Questionnaire (DHQ) II (18). The DHQ II is a food-frequency questionnaire of 134 food items that reports usual dietary intake over the past year and provides validated rank-order estimates of dietary intake (19), is recommended for assessment in older adults (20), and is commonly used with 1-year retrospective and prospective outcomes analyses. Nutrient and food intake data were quantified using the NCI's Diet^*^Calc software (21).

### High Glycemic Diet Measures

**Figure 2:**
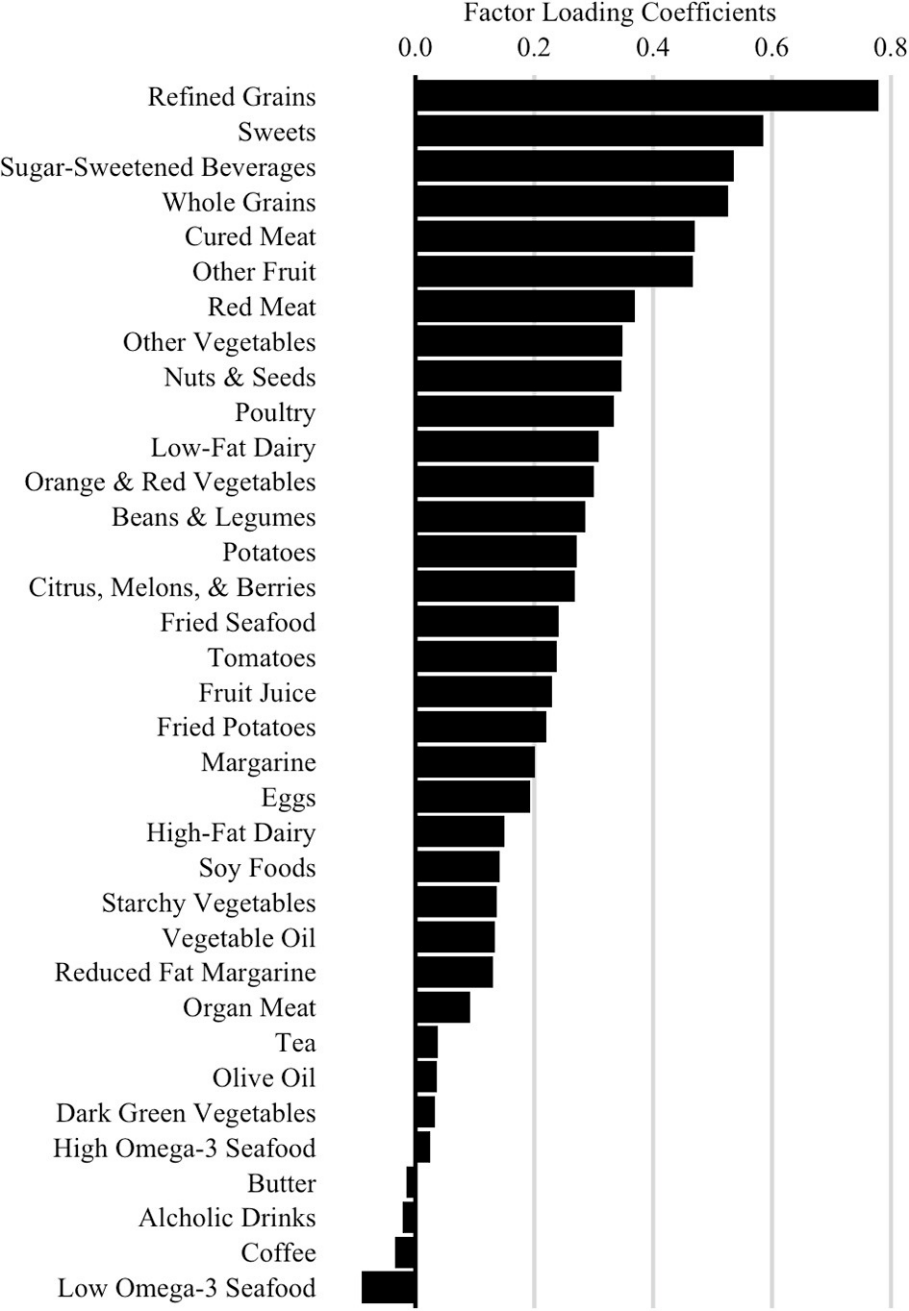
High Glycemic Diet (HGDiet) pattern factor loadings derived by principal covariates regression (PCovR). Factor loadings are represented as the bars (*n* = 102). High intake of foods that have high positive loadings resulted in higher HGDiet adherence scores. Conversely, low intake of foods with high positive loadings resulted in lower HGDiet adherence scores. The HGDiet pattern explained 10% of variation in dietary intake and 92% of variation in glycemic load.

Our primary dietary glycemic measure was a score representing adherence to a high glycemic diet (HGDiet) pattern derived using principal covariates regression (PCovR) to explain maximal variance in daily glycemic load consumption from the DHQ II output. We performed this using the [PCovR] (22) package for R (R Foundation, Vienna, Austria). We selected 35 food variables from the DHQ II, standardized the daily intake of each food variable to mean ± SD of 0 ± 1, and included them in the PCovR as independent variables to derive a component that we interpreted as the HGDiet pattern (23). We retained the first derived component which best explained variance in both dietary glycemic load (92%) and dietary intake (10%) using the varimax rotation method to scale the diet pattern. HGDiet pattern food loadings are presented in Figure 2, which included high positive loadings for foods with the highest contribution to the HGDiet pattern; namely, refined grains, sweets, and sugar-sweetened beverages. The HGDiet pattern also loaded highly on whole grains, cured meats, other fruit, and red meat. Individual adherence scores for the HGDiet pattern were calculated by multiplying each participant's standardized daily intake of each of the 35 food variables and summing the products of each of these foods with higher scores representing higher daily intake of the HGDiet pattern.

We also assessed glycemic load and daily intake of sugar and carbohydrate, standard output measures from the DHQII that represented sub-components of high glycemic diet. These variables were selected *a priori* based on our prior cross-sectional data suggesting a relationship with cerebral amyloid burden (11).

### APOE Genotype

Whole blood was collected and stored at −80C until we performed genetic analyses to determine APOE genotype using a Taqman single nucleotide polymorphism (SNP) allelic discrimination assay (ThermoFisher). We distinguished APOE4, APOE3, and APOE2 alleles using Taqman probes at the two APOE-defining SNPs, rs429358 (C_3084793_20) and rs7412 (C_904973_10). Participants with ≥1 APOE4 allele were classified as “APOE4 carriers”.

### Amyloid PET

Amyloid PET scans were obtained on a GE Discovery ST-16PET/CT scanner after 370 MBq administration of florbetapir F-18. Baseline scans were used to determine eligibility for participation in the APEx trial. Completers of the clinical trial repeated amyloid PET scans at 52 weeks. PET scans were obtained approximately 50 min post-injection as two continuous PET brain frames lasting 5 min each. The PET frames were summed and attenuation corrected prior to interpretation. We used the MIMneuro Amyloid Workflow (verson 6.8.7, MIM Software Inc., Cleveland, OH, USA) to calculate flobetapir F-18 standard uptake value ratios (SUVR) referenced to the whole cerebellum in six cortical regions of interest (ROI) after two-phase image registration: first rigid registration followed by deformable registration to a common template (24, 25). SUVR values were averaged across the left and right hemispheres. The six ROIs included the anterior cingulate gyrus, posterior cingulate gyrus, precuneus, inferior medial frontal gyrus, lateral temporal lobe, and superior parietal lobe.

Participants were classified as “elevated” using a general global SUVR cutpoint of 1.1 with a visual “over-read” process that improves consistency of amyloid PET scan interpretations in the early detection of amyloid accumulation (24). Raters referenced the ROI SUVR quantities, reviewed the individual baseline florbetapir F-18 PET images, and examined map projections of amyloid burden to determine “elevated” to “non-elevated” status. Majority agreement among the raters (≥2 raters) determined participant amyloid status classification. Participants with sub-threshold status did not meet the criteria for elevated amyloid yet had a mean SUVR global value >1.0 (average of the six ROIs). This value represented the upper half of non-elevated individuals that screened for participation in the APEx study. 1-year delta SUVR scores were calculated for the global average and for each ROI.

We also prepared longitudinal imaging data for voxel-based analysis with the Computational Anatomical Toolbox 12 (CAT12 Version 12.6, C. Gaser, Structural Brain Mapping Group, Jena University Hospital, Jena, Germany: http://dbm.neuro.uni-jena.de/cat/) through Statistical Parametric Mapping version 12 (SPM12, Wellcome Trust Center for Neuroimaging, London, UK: http://www.fil.ion.ucl.ac.uk/spm/software/spm12/) that operate under Matlab (R2019b, the Mathworks, Natick, MA) on Mac. We normalized PET images using a 50% probability threshold for gray matter specificity, co-registered them to the MNI152 template, and used 8 x 8 x 8 mm full-width half-maximum Gaussian kernel to smooth PET SUVR images.

### Statistical Analyses

The primary aim of this study was to investigate the relationship between the HGDiet pattern and 1-year change in cerebral amyloid SUVR in 6 ROIs and globally. We subsequently tested whether sub-components of high glycemic diet (glycemic load, sugar, and carbohydrate) were related to 1-year change in cerebral amyloid SUVR. Continuous variables were expressed as mean ± SDs. Differences in participant characteristics between elevated and sub-threshold amyloid status groups were assessed using independent samples *t*-test. Linear mixed models (LMM) assessing amyloid SUVR values as a function of time using a random intercept for subject were used to test for differences between baseline and week 52 amyloid levels among the entire sample and in participants with elevated amyloid.

For our primary analysis, we constructed LMMs to test the relationship between high glycemic diet and 1-year change in amyloid SUVR in the entire sample (*n* = 102). We used a random intercept for subject to account for repeated cerebral amyloid SUVR values (baseline and 1-year) and amyloid SUVR variability across subjects at baseline. Analyses included continuous HGDiet pattern scores, time, and their interaction, with the interaction test term providing the test for HGDiet's relationship with change in amyloid SUVR over 1 year. We subsequently constructed LMMs substituting continuous values of the high glycemic diet sub-components in place of HGDiet pattern scores. All models were adjusted for fixed effects of age, sex, group assignment, APOE4 status, and baseline BMI.

We also used voxel-based analyses to illustrate the regional relationship between HGDiet pattern adherence and amyloid accumulation over 1 year. To do this, we split HGDiet pattern scores at the median to create a binary “high” and “low” HGDiet variable and performed a longitudinal voxel-based 2 x 2 mixed ANOVA analysis on the normalized, smoothed cerebellar-standardized PET images using the “Flexible Factorial” model in CAT12, including age, sex, APOE carrier, and exercise group as covariates. *P*-values were corrected with family-wise error (FWE) for multiple comparisons. We created a visual statistical map using small volume correction (SVC) of the left and right precuneus from the integrated automatic anatomic labeling tool (26).

We also performed sensitivity analyses restricted to participants with elevated amyloid at enrollment (*n* = 70) investigating the relationship between high glycemic diet and change in amyloid SUVR over 1 year. To do this, we constructed LMMs similar to our primary analyses using only participants with elevated amyloid at baseline. We also constructed ordinary least squares (OLS) interaction models to assess the difference in relationship between the HGDiet pattern with amyloid SUVR change by group assignment (intervention vs. control) amyloid status at enrollment (elevated vs. sub-threshold), and APOE4 status (carrier or non-carrier). Randomization group interaction models were controlled for age, sex, APOE4 status, baseline BMI, and baseline region-specific SUVR values. APOE interaction models were controlled for age, sex, group assignment, baseline BMI, and baseline region-specific SUVR values.

Variation inflation factors were ≤ 1.1 for all models, indicating no covariate multicollinearity issues. Energy intake was considered as a potential covariate, but was excluded due to being highly colinear with our *a priori* high glycemic diet measures and having no relationship with cerebral amyloid. We assessed model assumptions using residual analyses including QQ plots, residual histograms, and scale-location plots. Statistical analyses were performed using R (v. 3.6.1; R Foundation, Vienna, Austria). Statistical significance was set at *p* < 0.05.

## Results

Data from 102 APEx study completers aged 65 to 88 years (70.1 ± 5.3) were available for analyses (Figure 1). As the primary study found no exercise related effect on amyloid (13), we combined participants from both the exercise (*n* = 70) and control (*n* = 32) groups in our analyses and adjusted the statistical models for group assignment and other *a priori* covariates. Baseline and 1-year change characteristics among all participants and amyloid status at enrollment (elevated vs. sub-threshold) are presented in Table 1. Individuals with elevated amyloid status at baseline were older (*p* = 0.02) and had higher SUVR change scores (indicator of more aggregated amyloid) in the inferior medial frontal gyrus (*p* = 0.01), lateral temporal lobe (*p* = 0.001), posterior cingulate gyrus (*p* = 0.01), precuneus (*p* = 0.02), and globally (*p* = 0.01) compared to the sub-threshold amyloid group. Not presented, the aerobic exercise group had a higher BMI at baseline (*p* = 0.04) and a larger increase in VO2 max (*p* = 0.002) than the control group. There were no characteristic differences between APOE4 carriers and non-carriers.

**Table 1:** Participant Characteristics^a^.

	**All (*n* = 102)**	**Elevated amyloid (*n* = 70)**	**Sub-threshold amyloid (*n* = 32)**	***P***
Age at enrollment	71.1 ± 5.3^b^	71.9 ± 5.5	69.3 ± 4.7	0.02
**Sex (F/M)**, ***n***	69/33	48/22	21/11	0.95
F/M, %	68/32	69/31	66/34	
**Race, ethnicity**, ***n*** **(%)**				0.23
White, non-hispanic	99 (97%)	69 (98%)	30 (94%)	
Black, non-hispanic	3 (3%)	1 (2%)	2 (6%)	
BMI at enrollment, kg/m^2^	28.4 ± 6.2	27.6 ± 5.2	30.3 ± 7.6	0.08
BMI change over 1 year, kg/m^2^	−0.3 ± 1.0	−0.3 ± 0.9	−0.4 ± 1.3	0.60
Fasting glucose at enrollment, mg/dL	99.4 ± 12.8	99.7 ± 13.3	98.7 ± 11.7	0.68
APOE4 carrier, *n* (%)	48 (47%)	37 (53%)	11 (34%)	0.13
Change in VO2 Max	1.5 ± 2.6	1.3 ± 2.3	1.9 ± 3.0	0.31
**Group randomization**				0.57
Intervention	70 (69%)	47 (67%)	24 (75%)	
Control	32 (31%)	23 (33%)	8 (25%)	
**Dietary intake^c^**				
Energy, kcal/day	1,630 ± 640	1,600 ± 610	1,680 ± 700	0.60
Fat, g/day	70 ± 33	68 ± 33	74 ± 33	0.37
Protein, g/day	68 ± 31	65 ± 29	72 ± 34	0.33
**Dietary glycemic measures^c^**				
HGDiet pattern	0.0 ± 1.0	0.0 ± 1.0	0.0 ± 1.0	0.93
Carbohydrate, g/day	183 ± 74	184 ± 72	182 ± 78	0.87
Sugar, g/day	84 ± 43	87 ± 45	80 ± 39	0.47
Glycemic load^d^	94 ± 41	93 ± 34	96 ± 47	0.79
**Baseline SUVR^e^**				
Global	1.21 ± 0.17	1.28 ± 0.16	1.05 ± 0.04	<0.001
Anterior cingulate gyrus	1.29 ± 0.21	1.38 ± 0.20	1.12 ± 0.07	<0.001
Inferior medial frontal gyrus	1.14 ± 0.18	1.21 ± 0.18	0.99 ± 0.05	<0.001
Lateral temporal lobe	1.23 ± 0.17	1.30 ± 0.17	1.07 ± 0.05	<0.001
Posterior cingulate gyrus	1.18 ± 0.17	1.25 ± 0.16	1.04 ± 0.08	<0.001
Precuneus	1.28 ± 0.22	1.38 ± 0.20	1.07 ± 0.07	<0.001
Superior parietal lobe	1.13 ± 0.17	1.19 ± 0.17	0.99 ± 0.08	<0.001
**1-Year SUVR change^e^**				
Global	0.009 ± 0.056	0.017 ± 0.061	–0.010 ± 0.039	0.01
Anterior cingulate gyrus	<0.001 ± 0.072	0.007 ± 0.078	–0.012 ± 0.054	0.16
Inferior medial frontal gyrus	0.007 ± 0.061	0.016 ± 0.065	–0.013 ± 0.045	0.01
Lateral temporal lobe	0.013 ± 0.064	0.026 ± 0.064	–0.015 ± 0.053	0.001
Posterior cingulate gyrus	0.006 ± 0.064	0.016 ± 0.069	–0.016 ± 0.047	0.01
Precuneus	0.020 ± 0.065	0.028 ± 0.073	0.003 ± 0.037	0.02
Superior parietal lobe	<0.005 ± 0.060	0.010 ± 0.065	–0.006 ± 0.044	0.14

a *Group differences assessed by independent samples t-test and Pearson's chi-square. Significance set at P < 0.05*.

b *Mean ± SD — all such values*.

c *Derived by the National Cancer Institute's Diet History Questionnaire II at study enrollment*.

d *Measure used to derive the High Glycemic Diet Pattern (HGDiet)*.

e *Derived using florbetapir F-18 positron emission tomography imaging at enrollment and end of study (1 year)*.

Supplementary Table 1 presents characteristic and dietary intake data from the high and low HGDiet adherence groups (determined by median split). Both groups had similar HEI-2015 diet quality scores (27). Relative to the low HGDiet adherence group, the high HGDiet adherence group was comprised of fewer females (57 vs. 78%), had higher energy and macronutrient intake (*p* < 0.001 for all), had higher sugar intake (111 ± 44 grams vs. 58 ± 19 grams), and had more precuneal amyloid accumulation (SUVR change of 0.034 ± 0.065 vs. 0.007 ± 0.064).

Among the entire sample (*n* = 102), amyloid SUVR values increased in the lateral temporal lobe (Δ = 0.013 ± 0.064, *p* = 0.04) and precuneus (Δ = 0.020 ± 0.065, *p* = 0.002) over 1 year. In participants with elevated amyloid at study enrollment (*n* = 70), amyloid SUVR values increased in the inferior medial frontal gyrus (Δ = 0.016 ± 0.065, *p* < 0.05), lateral temporal lobe (Δ = 0.026 ± 0.064, *p* = 0.001), precuneus (Δ = 0.028 ± 0.073, *p* = 0.002), and globally (Δ = 0.017 ± 0.061, *p* = 0.02). There were no changes in amyloid SUVR values in the sub-threshold group. Differences between baseline and week 52 amyloid SUVR values are presented in Supplementary Table 2.

### High Glycemic Diet and Amyloid Change

**Figure 3:**
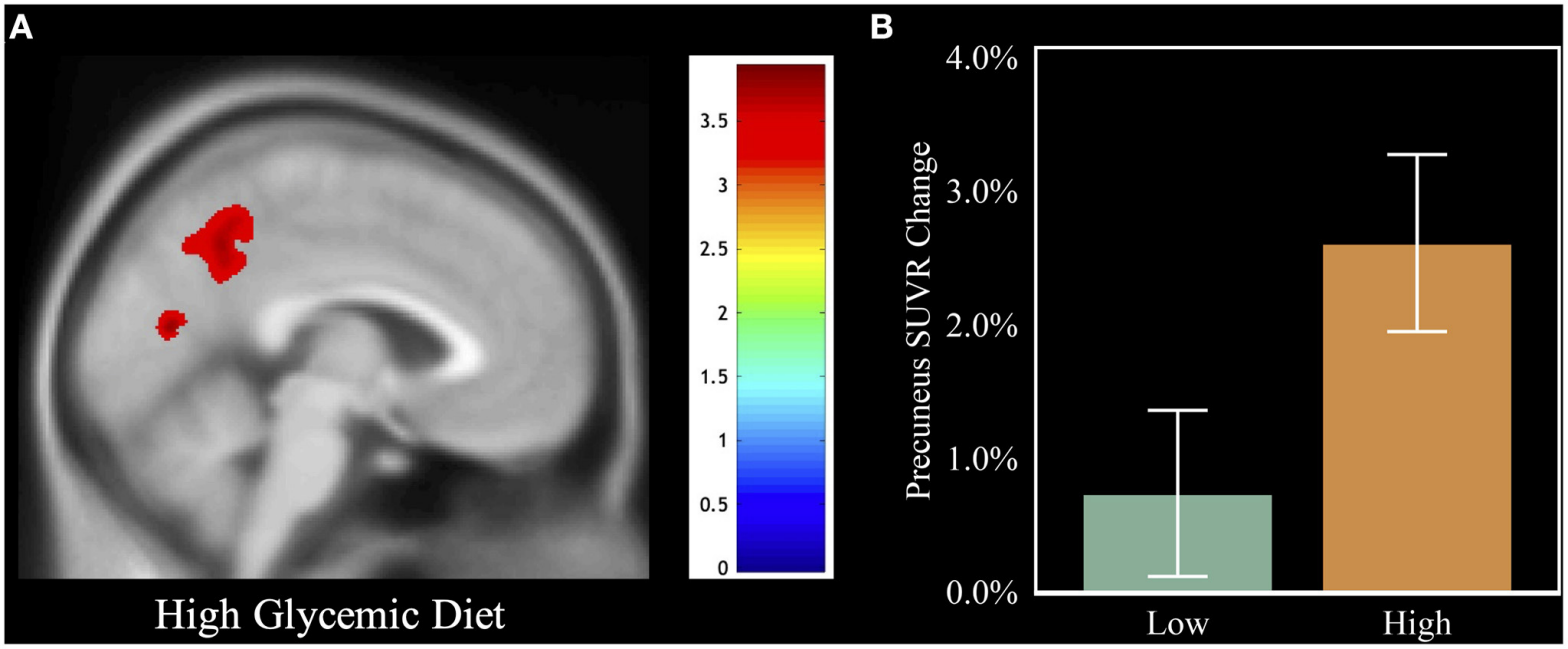
Visual of the relation between HGDiet intake and 1-year amyloid change. To illustrate the relation between HGDiet pattern scores and change in cerebral amyloid, we performed voxel-based amyloid PET analyses comparing SUVR change with a binary variable of adherence to the HGDiet pattern (high vs. low using a median split). **(A)** demonstrates that individuals in the high HGDiet group accumulated more amyloid over 1 year in the precuneus and cuneus compared to the low HGDiet group (*p* < 0.05 FWE corrected for multiple comparisons). **(B)** illustrates the contrast of mean precuneal amyloid accumulation between the high HGDiet and low HGDiet groups. Analyses were adjusted for age, sex, APOE, and treatment group.

We examined whether our primary (HGDiet) and secondary measures (glycemic load and daily intake of sugar and carbohydrate) correlated with change in amyloid in the 6 ROIs and the global average using LMM adjusted for age, sex, group assignment (exercise vs. control), APOE4 status, baseline BMI, and subject as a random effect (presented in Table 2 and Figure 3). For our primary analysis, higher intake of the derived HGDiet pattern was correlated with more amyloid accumulation in the precuneus (β = 0.06, *p* = 0.04). Likewise, our high glycemic diet sub-components of daily intake of sugar (β = 0.07, *p* = 0.01) and carbohydrate (β = 0.06, *p* = 0.04) were also correlated with more amyloid accumulation in the precuneus. Among the entire group, high glycemic diet measures were not related to amyloid change in the other ROIs nor globally.

**Table 2:** Relationship between high glycemic diet measures and regional change in cerebral amyloid burden among all participants^a^.

	**Primary**	**HGDiet sub-components**		
	**HGDiet pattern score**	**Glycemic load**	**Sugar (g)**	**Carbohydrate (g)**
**Regions with increasing SUVR over 1 year**				
Lateral temporal lobe	0.002 (−0.07–0.07)^a^	−0.02 (−0.09–0.05)	0.03 (−0.04–0.09)	0.003 (−0.07–0.07)
Precuneus	0.06 (0.0–0.11)^*^	0.03 (−0.02–0.09)	0.07 (0.01–0.12)^**^	0.06 (0.0–0.11)^*^
**Other regions of interest**				
Anterior cingulate gyrus	0.02 (−0.04–0.09)	0.002 (−0.06–0.07)	0.04 (−0.02–0.11)	0.02 (−0.04–0.09)
Inferior medial frontal gyrus	0.01 (−0.05–0.07)	−0.01 (−0.07–0.05)	0.03 (−0.03–0.10)	0.01 (−0.05–0.07)
Posterior cingulate gyrus	0.03 (−0.04–0.10)	0.02 (−0.05–0.09)	0.06 (−0.01–0.13)	0.04 (−0.03–0.11)
Superior parietal lobe	0.002 (−0.07–0.06)	−0.02 (−0.09–0.05)	0.01 (−0.06–0.08)	−0.004 (−0.07–0.06)
Global	0.02 (−0.04–0.09)	0.00 (−0.06–0.06)	0.04 (−0.02–0.11)	0.02 (−0.04–0.09)

a *Values are standardized β values (95% confidence intervals) for the interaction term of the respective dietary glycemic measure by time determined by linear mixed models. Linear mixed models were controlled for fixed effects of age, sex, group assignment, ApoE4 status (carrier/non-carrier), and BMI at baseline and a random effect of subject ID; n = 102*.

* *P < 0.05*,

** *P < 0.01*.

**Figure 4:**
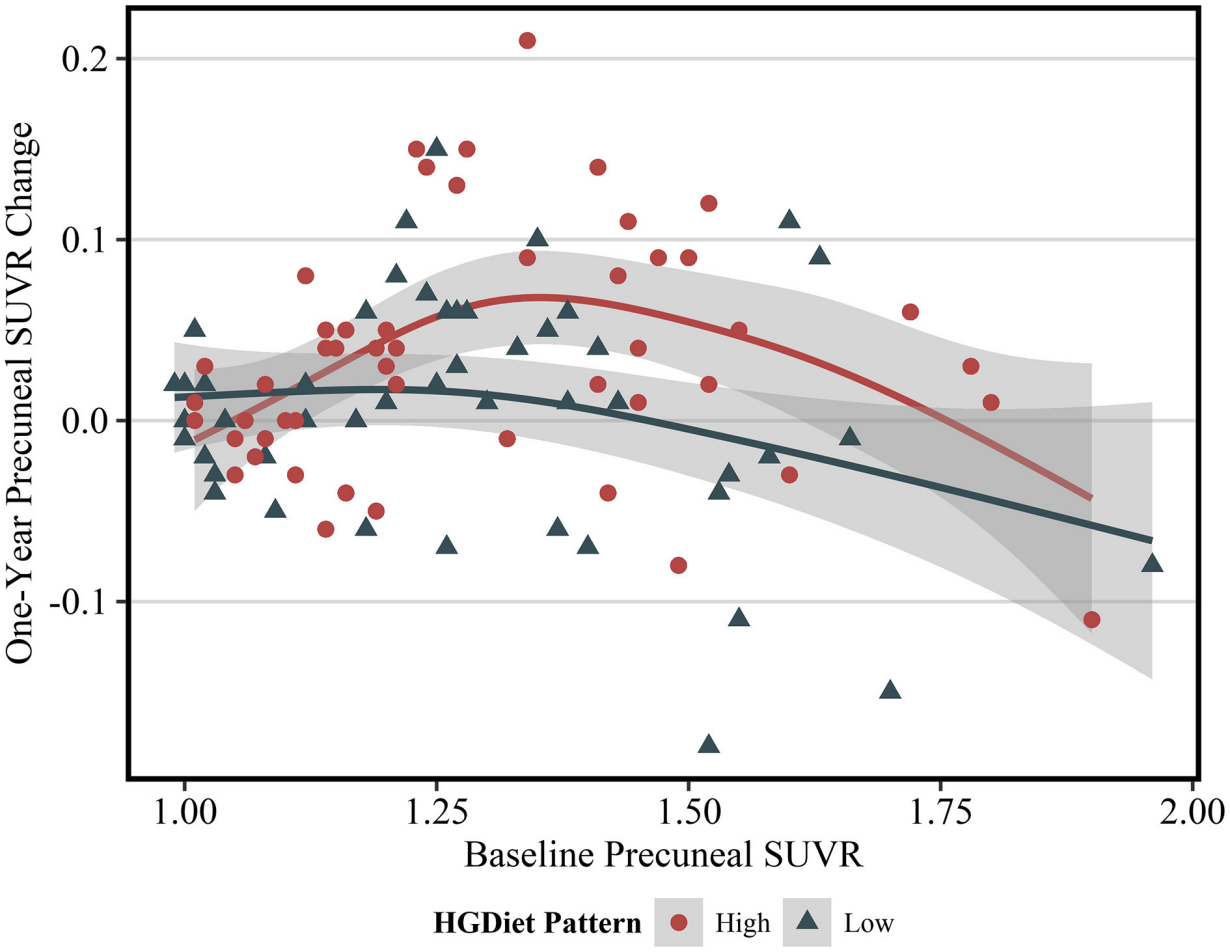
Scatterplot of 1 year change in PET SUVR values as a function of baseline PET SUVR in the precuneus region. The fit lines represent regression estimated means for categorical HGDiet pattern intake determined by a median split (High vs. Low). 1-year change in precuneal amyloid SUVR varied depending upon baseline amyloid SUVR values where those with moderately elevated amyloid status at baseline had the highest rate of amyloid accumulation over 1 year. HGDiet pattern adherence appeared to explain some of the variance in amyloid accumulation as a function of baseline levels with high HGDiet exhibiting an inverted U shape for rate of amyloid accumulation peaking higher than the more linear, downward trending fitment line for low HGDiet. Shaded areas are 95% confidence intervals.

Expected annual change in amyloid SUVR varies depending upon an individual's current amyloid load (28), thus we further investigated the observed relationship between the HGDiet pattern and amyloid accumulation in the precuneus by plotting 1-year precuneal amyloid SUVR change as a function of baseline precuneal SUVR values (Figure 4). Categorizing scatterplot points by either high or low adherence (median split) to the HGDiet pattern, amyloid accumulation in the high HGDiet group occurred on a parabolic curve trajectory (inverted U shape) with a higher peak than amyloid change in the low HGDiet group which occurred on a more linear decreasing trajectory. We performed a polynomial regression model of 1-year precuneal amyloid SUVR change as a function of the interaction between baseline precuneal amyloid SUVR and HGDiet (binomial) which indicated a difference in curvilinear fitment lines for trajectory of amyloid change between groups (*p* = 0.04).

### High Glycemic Diet and 1-Year Amyloid Change in Participants With Elevated Amyloid at Enrollment

**Figure 5:**
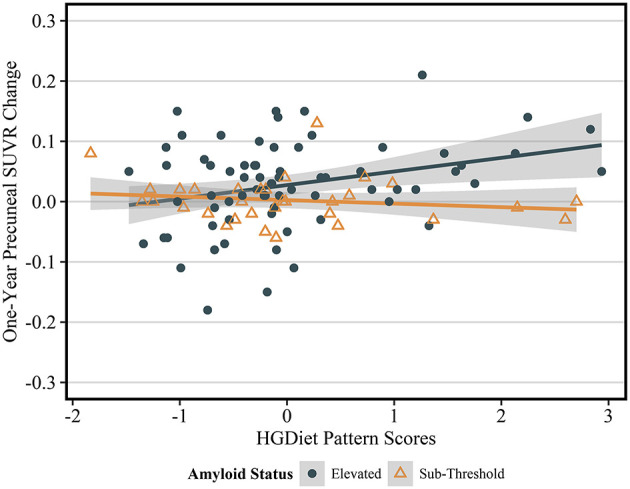
Sensitivity analysis of relationship between HGDiet pattern and 1-year change in precuneal amyloid stratified by baseline amyloid status. The relationship between HGDiet pattern scores and 1-year amyloid aggregation was strongest in the precuneus and accentuated in participants with elevated amyloid at baseline the (β = 0.40, *p* = 0.002). Beta- and *p*-values from ordinary least squares regression models were adjusted for age, sex, group assignment, APOE status, baseline BMI, and baseline precuneal SUVR value. Shaded areas are 95% confidence intervals. The relationship between HGDiet pattern scores and 1-year change in SUVR values was also accentuated in the lateral temporal lobe (β = 0.29, *p* = 0.02), posterior cingulate (β = 0.25, *p* = 0.03), and globally (β = 0.23, *p* = 0.02).

Because amyloid levels increased over 1 year in participants with elevated amyloid status at enrollment and were unchanged in participants with sub-threshold amyloid, we conducted sensitivity analyses testing the relationship between high glycemic diet and amyloid change only in participants with elevated amyloid status (Table 3). Higher intake of the HGDiet pattern was associated with more amyloid accumulation in the lateral temporal lobe (β = 0.09, *p* < 0.05), posterior cingulate gyrus (β = 0.09, *p* < 0.05), and precuneus (β = 0.11, *p* = 0.01) with nearly significant increase in global amyloid (β = 0.08, *p* = 0.07). Higher daily sugar and carbohydrate intake were also related to more amyloid accumulation in the posterior cingulate gyrus (sugar: β = 0.10, *p* < 0.05; carbohydrate: β = 0.10, *p* < 0.05) and precuneus (sugar: β = 0.11, *p* = 0.01; carbohydrate: β = 0.11, *p* = 0.01). Figure 5 further demonstrates the relationship between HGDiet scores and precuneal amyloid SUVR change in the elevated amyloid group.

**Table 3:** Sensitivity analysis restricted to participants with elevated amyloid at baseline examining relationship between high glycemic diet measures and regional change in cerebral amyloid burden^a^.

	**Primary**	**HGDiet sub-components**		
	**HGDiet pattern score**	**Glycemic load**	**Sugar (g)**	**Carbohydrate (g)**
**Regions with increasing SUVR over 1 year**				
Inferior medial frontal gyrus	0.06 (−0.03–0.14)^a^	0.03 (−0.06–0.12)	0.06 (−0.02–0.15)	0.05 (−0.03–0.14)
Lateral temporal lobe	0.09 (0.0–0.19)^*^	0.06 (−0.03–0.16)	0.09 (−0.01–0.18)^*†*^	0.08 (−0.01–0.17)
Precuneus	0.11 (0.03–0.19)^**^	0.08 (−0.01–0.16)^*†*^	0.11 (0.02–0.19)^**^	0.11 (0.02–0.19)^**^
Global	0.09 (−0.01–0.18)^*†*^	0.06 (−0.04–0.15)	0.08 (−0.01–0.18)^*†*^	0.08 (−0.01–0.17)^*†*^
**Other regions of interest**				
Anterior cingulate gyrus	0.07 (−0.02–0.17)	0.05 (−0.04–0.14)	0.08 (−0.01–0.17)	0.07 (−0.02–0.17)
Posterior cingulate gyrus	0.09 (0.0–0.19)^*^	0.08 (−0.02–0.18)	0.10 (0.0–0.19)^*^	0.10 (0.0–0.19)^*^
Superior parietal lobe	0.02 (−0.08–0.11)	−0.02 (−0.11–0.08)	0.01 (−0.08–0.11)	0.01 (−0.09–0.10)

a *Values are standardized β values (95% confidence intervals) for the interaction term of the respective dietary glycemic measure by time determined by linear mixed models. Linear mixed models were controlled for fixed effects of age, sex, group assignment, ApoE4 status (carrier/non-carrier), and BMI at baseline and a random effect of subject ID; n = 70*.

† *P < 0.08*,

* *P < 0.05*,

** *P < 0.01*.

### Interaction of High Glycemic Diet Pattern With Exercise and APOE

As this data came from a randomized controlled trial of exercise vs. controls, we examined interaction analyses of the role of group assignment (exercise vs. control) on the observed relationship between the HGDiet pattern and 1-year amyloid change. The relationships between the HGDiet pattern and change in amyloid over 1 year were the same in those assigned to exercise vs. control (interaction *p* = 0.20–0.87), which is not surprising given the trial found no effect of exercise on cerebral amyloid. We also performed interaction analyses for APOE status (carrier vs. non-carrier) groups, which did not influence the results (interaction *p* = 0.10–0.25). These models were controlled for age, sex, group assignment, APOE4 status, baseline BMI, and baseline SUVR values.

## Discussion

The current study corroborates our previous finding that a high glycemic diet is associated with increased amyloid burden in cognitively normal older adults (11) and extends that finding with longitudinal data suggesting a high glycemic diet may influence change in amyloid levels over the course of 1 year. The relationship between change in amyloid and high glycemic diet was apparent in the precuneus for the entire sample (*n* = 102). In only those with elevated amyloid status (*n* = 70), this relationship extended into the posterior cingulate and lateral temporal lobe, regions highly susceptible to amyloid accumulation and metabolic impairment due to AD-related pathology. This data suggests the need for further research to assess the causal nature of this observed relationship between diet and amyloid.

These longitudinal, observational data were derived from a randomized controlled trial (APEx) designed to determine the effect of aerobic exercise on amyloid burden in cognitively normal older adults with elevated amyloid. The main results of the APEx trial found no effect of 52 weeks of aerobic exercise on brain amyloid despite meaningful change in cardiorespiratory fitness (13). Our observation that a high glycemic diet is related to amyloid change over 1 year was similar in the exercise and non-exercise control group, suggesting that high glycemic diet may be more strongly related to amyloid levels than aerobic exercise. Although surprising, we believe the data is compelling in calling for further investigation given that we previously observed a relationship between high glycemic diet and amyloid burden at a single point in time (11) and now extend that observation to a high glycemic diet predicting some of the variance of amyloid accumulation over 1 year.

In addition to the longitudinal assessment from this study, there are important distinguishing characteristics of this analysis compared to the prior cross-sectional analysis (11). First, this study consisted of predominantly amyloid elevated participants (69%) whereas the prior cross-sectional analysis did not (26% amyloid elevated). Second, this study assessed change in amyloid over 1 year in a sample that was largely independent from our prior study, which included baseline SUVR data from 26% of participants represented in this study. Thus, the current study adds validity to our prior findings and suggests that high glycemic diet may influence amyloid accumulation in individuals that meet the definition for preclinical AD.

Our results are supported by other findings in the literature. AD is increasingly linked with metabolic disease (12) including impaired brain and peripheral glucose metabolism (29), which are measures tightly linked with diet. For instance, high glycemic diets cause sharp increases in peripheral blood glucose and insulin that are associated with chronic disease conditions (30), especially type two diabetes (31), a strong risk factor for AD-related neurodegeneration. We have previously linked elevated glucose levels with cerebral amyloid deposition (32) and at least one other study found that a high sugar diet (although combined with high fat) was associated with increased cerebral amyloid (33). Metabolic disruptions in energy flux required for protein homeostasis may alter processing of amyloid precursor protein and increase amyloid aggregation (10). As fasting hyperglycemia is increasingly acknowledged to increase AD risk and influence amyloid processing (32), it is also possible that postprandial glucose and insulin elevations due to chronic consumption of a high glycemic diet may elicit detrimental effects, even in the absence of fasting glucose abnormality.

The relationship between high glycemic diet and 1-year change in amyloid was primarily concentrated in the precuneus. In those with elevated amyloid status, there were also relationships in the lateral temporal lobe and posterior cingulate gyrus. These regions of the temporoparietal cortex have substantial metabolic demand requirements within the default mode network (DMN) (34), a highly active functional connection of brain regions during default state (rest) and memory retrieval (35). The precuneus remains metabolically active in the default state and during task performance due to its unique ability to switch network connectivity (36, 37). The temporoparietal regions are the earliest to exhibit amyloidosis and acute hypermetabolism in those with preclinical AD (38) and as amyloid further accumulates, severe metabolism decline is observed in these regions (38–41). Amyloid accumulation over the lifespan follows sigmoid curve trajectory where accumulation is slow in early life, speeds up in the transition phase, and once again slows in the saturation phase (42, 43). According to this curve, in individuals with preclinical AD and clinical dementia, annual average increases in amyloid SUVR depend upon baseline amyloid SUVR levels and increase between ~ 0.05 and 0.09 units during the most rapid phase of amyloid accumulation (28). Our data reflects the projected curve and suggests that in those most vulnerable for rapidly accumulating amyloid, higher intake of the HGDiet pattern may partially explain higher aggregation in amyloid over 1 year and lower intake of HGDiet may be protective. The unique nature of highly metabolic DMN regions, especially the precuneus, may make them susceptible to high glycemic diet's potential influence on peripheral and cerebral metabolism and dysregulation of amyloid processing.

Total reported sugar intake had the strongest relation with amyloid change in the precuneus and may be one of the most important components of a high glycemic diet. While there are no recommendations for total daily sugar intake in the US, the American Heart Association's recommendations for intake of added sugars are no more than 24 and 36 grams per day for most women and men, respectively (44). Mean intake of added sugars, sugars that are added to foods during processing, among the entire sample was 37.7 ± 24.5 grams and 50.1 ± 27.1 grams in those with the highest consumption of the HGDiet pattern. In this cohort, added sugars alone were not related to 1-year change in amyloid. Our total sugar assessment included a cumulative intake of all types of sugar, which have varying glycemic properties (45). Participants that gained new precuneal amyloid reported consuming an average of 91.0 ± 48.6 grams of sugar while those that either had reduced or stable precuneal amyloid consumed an average of 71.0 ± 31.8 grams of sugar. This suggests that comprehensive sugar intake may be most important when considering sugar's relation with AD risk. Additionally, the HGDiet pattern from this analysis loaded highly on sugar sweetened beverages, of which high intake is associated with low total brain volume (46) and may contribute to the detrimental relationship observed between HGDiet and cerebral amyloid.

High glycemic diet's relation with longitudinal change in brain amyloid provides a possible underlying mechanistic explanation for diets with purported reduction of AD risk. Healthy diets often promote lower consumption of refined carbohydrates and added sugars, although many vary in their recommended carbohydrate intake. For instance, the American Heart Association recommends a low-fat diet for cardiovascular health with 50–60% of energy as carbohydrate (47) while the Mediterranean diet, which is still carbohydrate rich, tends to have slightly higher fat and lower carbohydrate due to recommended consumption of olive oil, nuts, and fatty fish (48). Both diets have putative AD risk reduction benefits which may be driven by reduction of sugar and glycemic load, among other potential mechanisms. Nutrient-rich, ketogenic diets that reduce carbohydrate intake to ≤ 10% of energy as carbohydrate have also gained interest as a potential therapy for patients with AD (49–51). The ketone bodies generated by ketogenic diets are reported to have amyloid clearing properties within experimental *in vitro* and animal models (52, 53). The data from this analysis provides evidence to support future investigation into whether diet interventions that reduce sugar and glycemic load or outright carbohydrate restriction are capable of reducing amyloid burden in asymptomatic older adults with elevated amyloid.

There are strengths and limitations to consider from this study. This study analyzed data from 102 well-characterized, underactive older adults who were enrolled in a randomized clinical trial of exercise. The data thus could be confounded by the trial, however, exercise did not influence amyloid and all analyses are controlled for group assignment (exercise vs. control). Dietary habits were only assessed at baseline using the DHQII food frequency questionnaire which relies upon self-reported usual frequency and portion of food intake and is subject to potential over- or under-estimation of intake as well as selective reporting bias. Although we did not assess for possible changes in dietary habits during the study, this study did not include intervention to alter diet, thus it is unlikely that participants made large dietary changes. The longitudinal florbetapir F-18 PET scans allowed for assessment of 1-year change in cerebral amyloid, providing evidence that high glycemic diet not only correlates with cross-sectional amyloid burden, but also 1-year change. The study results do require caution in interpretation as this is observational evidence from a trial designed to investigate the effect of aerobic exercise on amyloid burden and does not represent a causal finding. This analysis also included a sample of mostly non-Hispanic white females, which limits the generalizability of our findings. Well-designed clinical trials with strict dietary protocol and sensitive dietary assessment tools will be necessary to determine whether there is a causal relationship between high glycemic diet and amyloid deposition.

Our data provides further support that diet is a modifiable behavior that may influence cerebral amyloid. Future studies are needed to determine whether there is indeed a causal relationship between high glycemic diet and brain amyloid accumulation and to rigorously test our hypothesis that high glycemic diet's relationship with amyloid is mediated by systemic metabolic effects. As AD prevalence in the United States is projected to double over the next 30 years (54), effective prevention and disease modifying therapies are urgently needed. Mounting evidence points to a role for diet and warrants strong investigational investment.

## Data Availability Statement

The raw data supporting the conclusions of this article will be made available by the authors, without undue reservation.

## Ethics Statement

The studies involving human participants were reviewed and approved by Institutional Review Board at the University of Kansas Medical Center. The patients/participants provided their written informed consent to participate in this study.

## Author Contributions

MT, DS, and JB designed the research. MT performed statistical analyses. RH and JDM provided statistical support. MT and JB drafted the manuscript. DS, JKM, EV, RH, and JDM provided critical revision for important intellectual content. MT had full access to the study data and takes responsibility for the final content of the manuscript. All authors read and approved the final manuscript.

## Funding

This research was supported by the National Institutes of Health (P30 AG035982, R01 AG043962, K01 AG065487, and R00 AG050490). Space, nursing and assay support were provided by UL1 TR000001. Avid Radiopharmaceuticals (Lilly) provided a grant to support Amyvid (florbetapir F-18) doses and PET scanning costs. Additional support was provided by the University of Kansas Medical Center Department of Dietetics and Nutrition.

## Conflict of Interest

The authors declare that the research was conducted in the absence of any commercial or financial relationships that could be construed as a potential conflict of interest.

## Publisher's Note

All claims expressed in this article are solely those of the authors and do not necessarily represent those of their affiliated organizations, or those of the publisher, the editors and the reviewers. Any product that may be evaluated in this article, or claim that may be made by its manufacturer, is not guaranteed or endorsed by the publisher.
