# Supplementary material for: High Glycemic Diet Is Related to Brain Amyloid Accumulation Over One Year in Preclinical Alzheimer's Disease
Source: Front Nutr. 2021 Sep 27;8:741534. doi: 10.3389/fnut.2021.741534 (PMC8502814; doi:10.3389/fnut.2021.741534)
Supplement: Supplementary file 1 [file Table_1.PDF]

Supplemental Table 1. Participant characteristics by binary split of HGDiet pattern scores. [1]

	High (n=51)	Low (n=51)	P
Age at enrollment	70.7 ± 5.7	71.4 ± 5.0	0.57
Sex (F/M), n	29/22	40/11	0.03
F/M, %	57/43	78/22	
BMI at enrollment, kg/m^2	29.4 ± 7.8	27.5 ± 3.9	0.14
APOE4 Carrier, n (%)	25 (49.0%)	23 (45.1%)	0.84
Fasting glucose at enrollment, mg/dL	100.5 ± 14.1	98.2 ± 11.3	0.37
Change in V02 Max	1.7 ± 2.4	1.3 ± 2.7	0.52
Group Randomization			0.20
Control	12 (23.5%)	19 (37.3%)	
Intervention	39 (76.5%)	32 (62.7%)	
Baseline Amyloid Status			1.0
Elevated	35 (69%)	35 (69%)	
Sub-Threshold	16 (31%)	16 (31%)	
Dietary Intake [3]			
Energy, kcal	2000 ± 650	1250 ± 300	<0.001
Fat, g	85 ± 37	55 ± 18	<0.001
Carbohydrate, g	234 ± 70	132 ± 31	<0.001
Protein, g	81 ± 33	54 ± 20	<0.001
Sugar, g	111 ± 44	58 ± 19	<0.001
Added Sugars, g	50.1 ± 27.1	25.3 ± 13.0	<0.001
Glycemic Load [4]	121 ± 39	67 ± 18	<0.001
HGDiet Pattern	0.7 ± 0.9	-0.7 ± 0.4	<0.001
HEI-2015	67.8 ± 8.4	67.4 ± 9.4	0.45
Total Fruit	4.1 ± 1.4	3.7 ± 1.4	0.13
Whole Fruit	4.6 ± 1.1	4.5 ± 1.0	0.73
Total Vegetables	4.2 ± 1.0	4.2 ± 1.0	0.70
Greens and Beans	3.8 ± 1.6	4.1 ± 1.5	0.32
Whole Grains	3.0 ± 1.8	2.8 ± 1.6	0.47
Dairy	6.1 ± 2.5	5.7 ± 2.6	0.42
Total Protein Foods	4.7 ± 0.6	4.7 ± 0.8	0.59
Seafood and Plant Proteins	4.8 ± 0.7	4.5 ± 1.1	0.18
Fatty Acids	6.0 ± 2.7	6.1 ± 2.9	0.81
Refined Grains	8.4 ± 1.9	8.5 ± 1.9	0.88
Sodium	4.9 ± 2.7	4.2 ± 2.9	0.21
Added Sugars	7.2 ± 2.0	8.7 ± 2.1	0.03
Saturated Fats	6.0 ± 2.6	5.7 ± 2.7	0.50
Baseline SUVR [5]			
Anterior Cingulate Gyrus	1.29 ± 0.22	1.30 ± 0.20	0.77
Inferior Medial Frontal Gyrus	1.14 ± 0.19	1.14 ± 0.17	0.84
Lateral Temporal Lobe	1.24 ± 0.19	1.21 ± 0.15	0.33
Posterior Cingulate Gyrus	1.20 ± 0.16	1.17 ± 0.18	0.44
Precuneus	1.29 ± 0.22	1.27 ± 0.23	0.63
Superior Parietal Lobule	1.14 ± 0.17	1.11 ± 0.18	0.43
One-Year SUVR Change [5]			
Anterior Cingulate Gyrus	0.007 ± 0.073	-0.006 ± 0.071	0.34
Inferior Medial Frontal Gyrus	0.012 ± 0.062	0.002 ± 0.059	0.41
Lateral Temporal Lobe	0.018 ± 0.073	0.009 ± 0.053	0.49
Posterior Cingulate Gyrus	0.012 ± 0.072	<0.001 ± 0.055	0.33
Precuneus	0.034 ± 0.065	0.007 ± 0.064	0.03
Superior Parietal Lobule	0.008 ± 0.062	0.002 ± 0.058	0.63

[1] Group differences assessed by independent samples t-test and Pearson's chi-square. Significance set at P<0.05.
[2] Mean ± SD – all such values.
[3] Derived by the National Cancer Institute's Diet History Questionnaire II at study enrollment.
[4] Measure used to derive the High Glycemic Diet Pattern (HGDiet)
[5] Derived using florbetapir F-18 positron emission tomography imaging at enrollment and end of study (1 year).
