# Supplementary material for: High Glycemic Diet Is Related to Brain Amyloid Accumulation Over One Year in Preclinical Alzheimer's Disease
Source: Front Nutr. 2021 Sep 27;8:741534. doi: 10.3389/fnut.2021.741534 (PMC8502814; doi:10.3389/fnut.2021.741534)
Supplement: Supplementary file 2 [file Table_2.PDF]

Supplemental Table 2. One-year change in amyloid SUVR among all participants and restricted to those with elevated amyloid. [1]

	All Participants (n=102)			Elevated Amyloid (n=70)		
	Baseline	Week 52	P	Baseline	Week 52	P
Global	1.206 ± 0.172 [2]	1.214 ± 0.178	0.12	1.283 ± 0.158	1.300 ± 0.150	0.02
Anterior Cingulate Gyrus	1.292 ± 0.206	1.293 ± 0.212	0.92	1.376 ± 0.197	1.382 ± 0.197	0.484
Inferior Medial Frontal Gyrus	1.136 ± 0.180	1.143 ± 0.189	0.26	1.208 ± 0.177	1.223 ± 0.177	0.05
Lateral Temporal Lobe	1.221 ± 0.176	1.234 ± 0.183	0.04	1.296 ± 0.166	1.322 ± 0.155	0.001
Posterior Cingulate Gyrus	1.182 ± 0.169	1.188 ± 0.186	0.35	1.249 ± 0.160	1.265 ± 0.170	0.06
Precuneus	1.278 ± 0.223	1.298 ± 0.227	0.002	1.378 ± 0.203	1.406 ± 0.194	0.002
Superior Parietal Lobe	1.124 ± 0.175	1.129 ± 0.174	0.39	1.192 ± 0.169	1.202 ± 0.155	0.197

[1] Group differences assessed by linear mixed models for amyloid SUVR values as a function of time. Linear mixed models included the random effect of subject ID. Significance set at $P<0.05$.
[2] Mean ± SD – all such values.
